# Role of biomarkers in predicting mortality in patients with flaviviral disease endemic to South India: a retrospective observational study

**DOI:** 10.1186/s12879-025-11509-x

**Published:** 2025-09-26

**Authors:** Nitin Gupta, Pothumarthy Venkata Swathi Kiran, Mohammad Khalid, Tirlangi Praveen Kumar, Prithvishree Ravindra, Rachana Bhat

**Affiliations:** 1 Department of Infectious Diseases, Kasturba Medical College, Manipal, Manipal Academy of Higher Education, Manipal, India; 2 Department of Emergency Medicine, Kasturba Medical College, Manipal,Manipal Academy of Higher Education, Manipal, India

**Keywords:** Flavivirus, Dengue, Kyasanur forest disease, Biomarker

## Abstract

**Background:**

Flaviviral infections such as dengue and Kyasanur Forest Disease (KFD) are endemic causes of acute febrile illness in South India, with some cases progressing to severe disease and death.

**Methods:**

We conducted a retrospective observational study at a tertiary care centre from June 2023 to June 2024, enrolling 107 adults diagnosed with dengue (NS1/IgM positive) or KFD (Polymerase chain reaction assay-confirmed). Clinical and biomarker data, including haematological, hepatic, renal, inflammatory, and endothelial parameters, were analysed to find differences between the two diseases and identify predictors of 28-day mortality.

**Results:**

Eleven patients (10.3%) died within 28 days. KFD patients were older and had higher adrenomedullin and transaminase levels. Dengue cases showed higher SOFA scores and more severe thrombocytopenia. Mortality was associated with elevated coagulation markers, serum creatinine, procalcitonin, and adrenomedullin. Aspartate aminotransferase was more frequently elevated in non-survivors.

**Conclusions:**

Combining conventional and novel biomarkers, such as adrenomedullin, may help early identification of high-risk patients, improving prognostication and management of endemic flaviviral infections.

## Introduction

Flaviviral infections such as dengue and Kyasanur Forest Disease (KFD) are endemic to South India and are recognised causes of acute undifferentiated febrile illness (AUFI) in the region [1]. Dengue, transmitted by *Aedes* mosquitoes, is the most common arboviral infection in India, with periodic outbreaks placing a significant burden on healthcare systems [2]. KFD, a tick-borne viral hemorrhagic fever endemic to forested regions of Karnataka, Kerala, Tamil Nadu, Maharashtra and Goa [2]. In endemic areas, it is associated with a significant burden, especially in the dry season. Both diseases are very similar in presentation, with high-grade fever, leucopenia, and thrombocytopenia, and it is often difficult to differentiate between the two, especially in primary care settings [3]. Although the majority of patients with dengue or KFD experience a self-limiting illness, a subset may develop complications such as bleeding, liver dysfunction, encephalitis, renal impairment, or shock [4]. The clinical progression is often rapid, and timely recognition of severe disease is essential to initiate supportive care and refer to facilities with intensive monitoring support. Biomarkers, measurable indicators of biological or pathological processes, offer a promising avenue to enhance risk stratification. This study, therefore, aimed to evaluate the proportion of patients who die within 28 days and the prognostic value of a panel of biomarkers in predicting mortality in adult patients with endemic flaviviral infections.

## Methodology

We conducted a retrospective observational study at a tertiary care centre in South India between June 2023 and June 2024 after taking approval from the Institute’s ethical approval committee. Adult patients (> 18 years) diagnosed with dengue (confirmed by NS1 antigen or IgM ELISA) or KFD (confirmed by polymerase chain reaction assay) were screened from hospital records for inclusion. The patients were recruited if details of clinical profile, biomarkers and outcomes were available. The implicit exclusion criteria were age < 18 years, unconfirmed diagnosis, or incomplete records.

The primary outcome was all-cause 28-day mortality. The pooled mortality in patients with dengue in a systematic review and meta-analysis was 5% [5]. The mortality in KFD patients in a study from Karnataka was 10%. Assuming the combined mortality of 7.5% for flaviviruses, with an envisioned precision of 5%, and a confidence level of 95%, a sample size of 107 was required.

Based on the primary outcome, patients were divided into survivors and non-survivors. The duration of illness was defined as the number of days from the onset of fever to the time of presentation at our tertiary care centre. The details of the following conventional laboratory investigations were noted: hematocrit, total leukocyte count, platelet count, prothrombin time/international normalized ratio (PT/INR), activated partial thromboplastin time (aPTT), C-reactive protein (CRP), serum creatinine, aspartate aminotransferase (AST), alanine aminotransferase (ALT), and erythrocyte sedimentation rate (ESR). The details of novel biomarkers such as adrenomedullin and procalcitonin were also collected, wherever feasible. In this study, certain variables were categorised based on established clinical thresholds or commonly used reference cut-offs. Thrombocytopenia was defined as a platelet count of < 50,000/µL, a level associated with increased bleeding risk in flaviviral infections [6]. Raised CRP was defined as > 6 mg/L (based on institutional cut-offs), and elevated procalcitonin as > 0.5 ng/mL (based on institutional cut-offs), both indicating significant systemic inflammation. Transaminitis was defined by AST or ALT levels > 10 times the upper limit of normal (ULN), consistent with severe hepatic involvement, as seen in other viral hepatitis [7]. ULN was considered as 50 IU/L for both AST and ALT. These categorisations allowed for simplified risk stratification and comparative analysis across groups. Other variables were analysed as continuous parameters to retain their full distribution and improve sensitivity in detecting associations with mortality.

Biomarker levels and clinical parameters were compared between patients with dengue and KFD, and also between patients who survived and those who did not. Categorical variables were analysed using the chi-square test or Fisher’s exact test. Continuous variables were analysed using the independent t-test or the Mann-Whitney U test, based on data distribution. A two-tailed *p*-value < 0.05 was considered statistically significant. The predictive performance of all biomarkers was evaluated using the Receiver Operating Characteristic (ROC) curve analysis. Those biomarkers found to have a statistically significant Area Under the Curve (AUC) were plotted. The optimal cutoff point for each significant biomarker was determined by identifying the value that maximised the Youden’s J statistic (Sensitivity + Specificity − 1).

## Results

Of the 107 patients included, 77 were diagnosed with dengue and 30 with Kyasanur Forest Disease (KFD). There were no cases of co-infection in our study cohort. As shown in Table 1, patients with KFD were significantly older (54.6 vs. 40 years, *p* < 0.001) and had a longer duration of illness at presentation (5.8 vs. 4.5 days, *p* = 0.004). Dengue patients had higher SOFA scores (median 4 vs. 3, *p* = 0.004), lower platelet counts (25 vs. 89 × 10³/µL, *p* < 0.001), and elevated CRP levels (14.5 vs. 1.4 mg/L, *p* < 0.001). In contrast, patients with KFD had significantly lower total leukocyte counts (2750 vs. 6150/µL, *p* < 0.001), higher AST (652 vs. 165 IU/L, *p* < 0.001) and ALT (312 vs. 86.3 IU/L, *p* = 0.002) levels, and markedly elevated adrenomedullin levels (729 vs. 95.1 pg/mL, *p* < 0.001). No significant differences were observed in INR, aPTT, creatinine, ESR, or procalcitonin levels between the two groups.

**Table 1:** The baseline characteristics of patients with dengue and patients with Kyasanur forest disease

Parameter	Reference range	Dengue (*n* = 77)	KFD (*n* = 30)	*p*-value
Age (years)	-	40 ± 13.6	54.6 ± 13.3	< 0.001
Duration of illness at presentation (days)	-	4.5 ± 1.8	5.8 ± 2.4	0.004
SOFA score	-	4 (3–5)	3 (2–4)	0.004
Platelet count (×10³/µL)	50–150	25 (13.25–55.25)	89 (62.4–118.5)	< 0.001
Total leukocyte count (/µL)	4000–11,000	6150 (3800–8850)	2750 (2400–3900)	< 0.001
INR	0.8–1.2	1.06 ± 0.27	1.03 ± 0.11	0.547
aPTT (seconds)	25–35	40.3 ± 10.63	41 ± 13.5	0.796
CRP (mg/L)	0–6	14.5 (5–28.7)	1.4 (0.6–14)	< 0.001
Creatinine (mg/dL)	0.6 to 1.2	0.95 (0.73–1.13)	0.92 (0.88–1.00)	0.074
AST (IU/L)	0–40	165 (82–322)	652 (241–700)	< 0.001
ALT (IU/L)	0–40	86.3 (51.3–195.5)	312 (198.5–507.5)	0.002
Adrenomedullin (pg/mL)	NA	95.1 (60–211)	729 (527–859)	< 0.001
ESR (mm/hr)	0–20	10.5 (2–24.7)	10 (2–17)	0.829
Procalcitonin (ng/mL)	0-0.5	0 (0–88)	0.8 (0–1.46)	0.376

Data are presented as mean ± SD or median (IQR), as appropriate

ESR and procalcitonin values were available for only 88 and 60 patients, respectively

*CRP* C-reactive protein, *INR* International Normalised Ratio, *aPTT* activated Partial Thromboplastin Time, *AST* Aspartate Aminotransferase, *ALT* Alanine Aminotransferase, *ESR* Erythrocyte Sedimentation Rate

A total of 11 patients (10.3%) died within 28 days of presentation. As shown in Table 2, non-survivors had a higher likelihood of a KFD diagnosis, significantly higher INR (1.3 vs. 1.02, *p* < 0.001), prolonged aPTT (50.7 vs. 39.3 s, *p* = 0.001), and elevated serum creatinine (1.72 vs. 0.87 mg/dL, *p* = 0.001). ADM levels were significantly higher in non-survivors (840 vs. 118 pg/mL, *p* = 0.037), and AST elevations > 10× ULN were more frequent (72.7% vs. 29.2%, *p* = 0.004). Elevated procalcitonin (> 0.5 ng/mL) was also more commonly observed in those who died (88.9% vs. 43.1%, *p* = 0.001). There were no significant differences in CRP, ESR, or ALT levels between survivors and non-survivors.

**Table 2:** Comparison of patients with flaviviral infection based on their 28-day mortality outcomes

Parameter		No Mortality (*n* = 96)	Mortality at 28 Days (*n* = 11)	*p*-value
Diagnosis	Dengue	72 (75%)	5 (45.5%)	**0.039**
	KFD	24 (25%)	6 (54.5%)	
Thrombocytopenia < 50 × 10³/µL (*n*, %)		60 (62.5%)	4 (36.4%)	0.094
Total leukocyte count (/µL)		3900 (2700–7150)	9600 (3200–13,250)	0.157
INR		1.02 ± 0.13	1.3 ± 0.6	**< 0.001**
aPTT (seconds)		39.3 ± 9.8	50.7 ± 18.7	**0.001**
Raised CRP > 6 mg/L (*n*/*N*, %)		32/51 (62.7%)	5/9 (55.6%)	0.683
Creatinine (mg/dL)		0.87 (0.73–1.08)	1.72 (1.07–3.27)	**0.001**
AST > 10× ULN (*n*, %)		28 (29.2%)	8 (72.7%)	**0.004**
ALT > 10× ULN (*n*, %)		15 (15.6%)	4 (36.4%)	0.088
Adrenomedullin (pg/mL)		118 (65–526)	840 (494–1172)	**0.037**
ESR (mm/hr)		10 (2–20.5)	11 (6–63)	0.291
Raised Procalcitonin > 0.5 ng/mL (*n*/*N*, %)		22/51 (43.1%)	8/9 (88.9%)	**0.001**

Data are presented as mean ± SD, median (IQR), or number (%) as appropriate. Bold *p*-values are significant

*KFD* Kyasanur Forest Disease, *INR* International Normalised Ratio, *aPTT* activated Partial Thromboplastin Time, *CRP* C-reactive protein, *AST* Aspartate Aminotransferase, *ALT* Alanine Aminotransferase, *ULN* Upper Limit of Normal, *ESR* Erythrocyte Sedimentation Rate

**Fig. 1:**
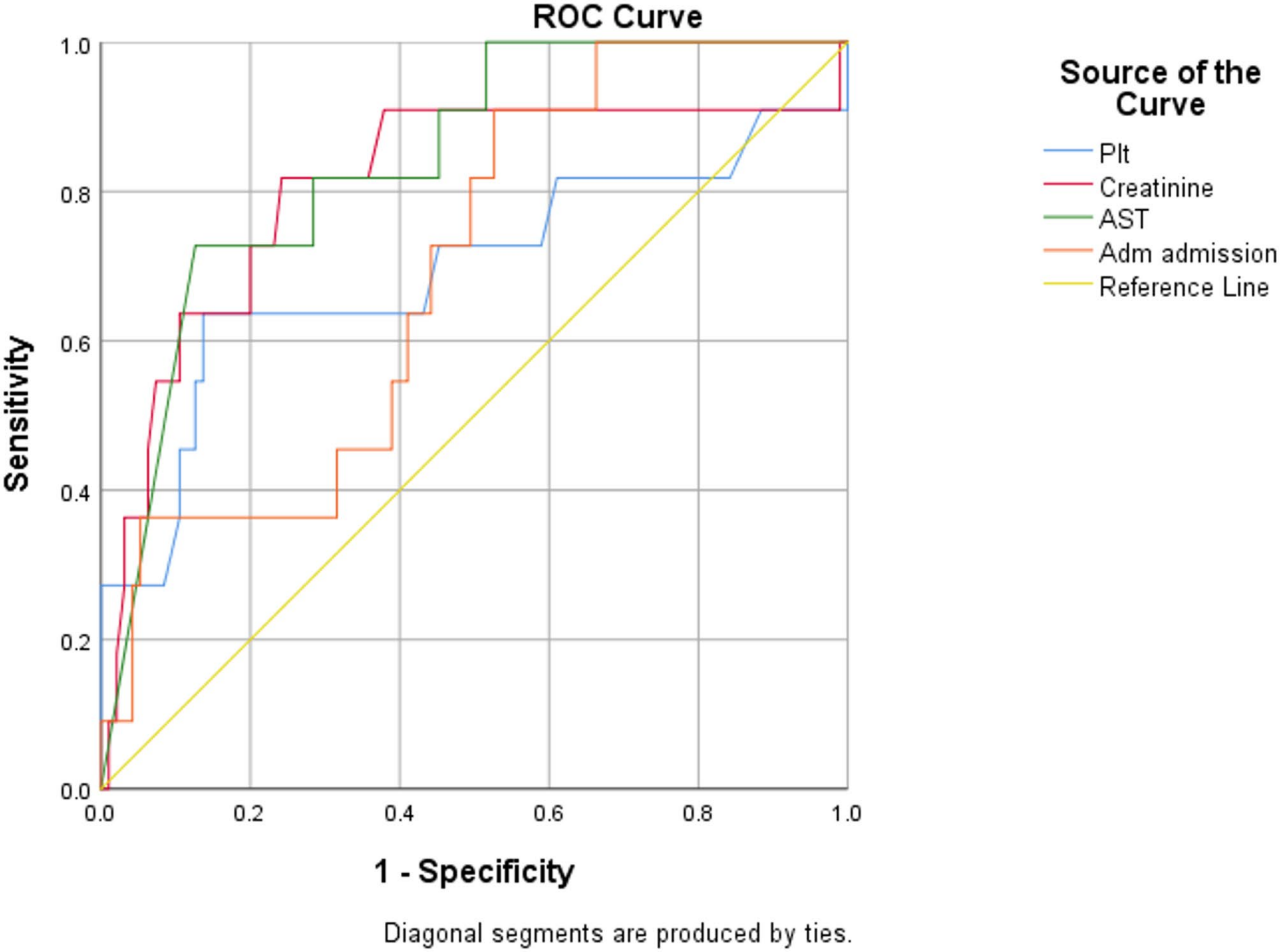
Receiver Operating Characteristic Curves for Predicting 28-Day Mortality. Abbreviation: Plt: Platelet count, AST: Aspartate Aminotransferase, Adm: Adrenomedullin at admission

AST, creatinine, platelet count, and adrenomedullin were found to have statistically significant AUC to predict 28-day mortality (Fig. 1). Among these, AST demonstrated the highest discriminatory ability with an AUC of 0.840, followed by creatinine with an AUC of 0.807 (Table 3). Platelet count and adrenomedullin also showed significant predictive power with AUCs of 0.694 and 0.693, respectively (Table 3).

**Table 3:** Receiver operating characteristic curve analysis of biomarkers for predicting 28-Day mortality

Variable	AUC (95% CI)	*p*-value	Optimal Cutoff	Sensitivity (%)	Specificity (%)
AST	0.840 (0.730–0.950)	< 0.001	> 695.5 IU/L	72.70%	87.40%
Creatinine	0.807 (0.641–0.974)	0.001	> 1.07 mg/dL	81.80%	70.50%
Platelet Count	0.694 (0.485–0.902)	0.036	≤ 91,000/µL	54.50%	86.30%
Adrenomedullin	0.693 (0.547–0.838)	0.037	> 833.8 pg/mL	27.30%	95.80%

*Abbreviation* *AUC *Area Under the Curve, *CI *Confidence Interval, *AST *Aspartate Aminotransferase

## Discussion

This study aimed to identify biomarkers in flaviviral illness that can predict 28-day mortality in adult patients with flaviviral infections, dengue and KFD, in a region where both are endemic. Among the 107 patients included, 11 (10.3%) died within 28 days. Mortality was significantly associated with deranged coagulation parameters (INR, aPTT), liver dysfunction (elevated AST), renal dysfunction (elevated creatinine), and higher levels of adrenomedullin and procalcitonin. ROC analysis confirmed that AST (AUC 0.840) and creatinine (AUC 0.807) were strong and statistically significant predictors of mortality. These findings suggest that a panel of routinely available and novel biomarkers can help identify patients at higher risk of poor outcomes, enabling timely triage and intervention.

There were notable baseline differences between patients with dengue and those with KFD. Patients with KFD were significantly older and presented later in the course of illness since most patients with KFD were referred to our hospital from neighbouring districts, whereas dengue cases came from within the district as well. While dengue patients had higher SOFA scores and more severe thrombocytopenia, KFD patients had significantly higher transaminase levels and markedly elevated adrenomedullin. CRP levels were also considerably lower in KFD, reflecting possible differences in the inflammatory response or endothelial involvement between the two flaviviral infections. These distinctions highlight the importance of recognising disease-specific clinical patterns, even within the broad category of tropical flaviviral infections.

Among the various parameters evaluated, several emerged as strong predictors of mortality in this cohort. Coagulation abnormalities, including raised INR and aPTT, are common in dengue and indicate severe disease. In a systematic review and meta-analysis, prolonged aPTT and INR were noted in 42% of the patients with dengue [8]. Prolongation of PT and aPTT in dengue is multifactorial, resulting from impaired hepatic synthesis of coagulation factors due to liver injury, and increased consumption of these factors during systemic inflammation and endothelial activation [9]. While dengue patients presented with more severe thrombocytopenia, our data showed that having thrombocytopenia was not significantly associated with 28-day mortality in the overall cohort. Additionally, the dengue virus NS1 protein may inhibit prothrombin activation, while cytokines like IL-6 downregulate factor XII synthesis, contributing to intrinsic pathway abnormalities [10]. In KFD, aPTT is more frequently prolonged when compared to PT, and this has been shown to be associated with higher mortality [4].

Notably, AST elevation of more than ten times the upper limit of normal was more common among non-survivors, suggesting hepatocellular injury as an important contributor to severe disease. In a systematic review, elevated levels of AST and ALT were consistently associated with more severe forms of dengue [11]. AST elevations were often greater than ALT due to additional release from non-hepatic sources like muscle and erythrocytes [11]. At the optimal cutoff of > 695.5 IU/L, AST predicted mortality with a sensitivity of 72.7% and a specificity of 87.4%, yielding an AUC of 0.840. Higher transaminase levels correlated with bleeding manifestations, thrombocytopenia, and hepatomegaly. AST levels > 1,000 IU/L were observed in severe dengue and could signal impending complications [11]. Similar findings have been noted in KFD as well [4].

Novel biomarkers like adrenomedullin and procalcitonin were also significantly elevated in patients who died, supporting their potential role as early prognostic indicators. Although procalcitonin has been traditionally viewed as a marker of bacterial infection, studies have shown its role in prognostication in febrile illnesses. A prospective study from Thailand found that elevated serum procalcitonin was independently associated with the development of dengue shock and/or organ failure [12]. At the optimal cutoff of > 833.8 pg/mL, adrenomedullin predicted mortality with a specificity of 95.8%, yielding an AUC of 0.693. Adrenomedullin is a vasoactive peptide with key roles in maintaining vascular tone, stabilizing the endothelium, and modulating inflammation [13]. In children with severe dengue, adrenomedullin levels were significantly higher than in healthy controls and correlated with markers of plasma leakage [13]. Levels were highest in fatal cases, suggesting that adrenomedullin may serve as a useful biomarker for predicting disease severity and endothelial dysfunction in dengue [13]. The high adrenomedullin levels likely reflect severe systemic involvement and a failing endothelial barrier, contributing to multi-organ dysfunction and death.

This study has several limitations. The statistical power to identify robust predictors of mortality was limited by the small number of non-survivors and the wide variance in biomarker levels observed within this group. This necessitates a cautious interpretation of these findings, which should be considered preliminary until validated in larger prospective studies. As a retrospective study focused on biomarkers, we did not systematically collect detailed clinical data on patient comorbidities, specific treatment modalities, or the exact causes of mortality, the absence of which may limit the interpretation of our findings, as these factors could be important confounders. Some biomarkers, such as procalcitonin and ESR, were not available for all patients, potentially introducing bias. The study was conducted at a single centre in South India, which may affect the generalizability of findings to other endemic regions. Also, as this was a retrospective study based on hospital records, the biomarkers were measured at the time of presentation and during hospitalisation as clinically indicated, rather than on a protocol-defined, uniform day of illness.

In conclusion, coagulation abnormalities, liver dysfunction, renal dysfunction, and raised levels of adrenomedullin and procalcitonin were associated with worse outcomes. These findings support the integration of such biomarkers into clinical workflows for the early identification of high-risk patients. Further multicentric studies with larger cohorts are warranted to validate these findings and to explore biomarker-guided intervention strategies in endemic settings.

## Data Availability

Data is provided within the manuscript. Any additional data will be provided by corresponding author on reasonable request.

## Abbreviations

KFD: Kyasanur Forest Disease
SOFA: Sequential organ failure assessment
AUFI: Acute undifferentiated febrile illness
